# Prediction of Simulated 1,000 m Kayak Ergometer Performance in Young Athletes

**DOI:** 10.3389/fpubh.2020.526477

**Published:** 2021-01-20

**Authors:** André B. Coelho, Fábio Y. Nakamura, Micaela C. Morgado, Francisco Alves, Angela Di Baldassarre, Andrew Flatt, Luis Rama

**Affiliations:** ^1^Faculty of Sports Science and Physical Education, Research Center for Sport and Physical Activity Centro de Investigação em Desporto e Atividade Física (CIDAF), University of Coimbra, Coimbra, Portugal; ^2^Portuguese Canoe Federation Team, Vila Nova de Gaia, Portugal; ^3^Department of Medicine and Aging Sciences, “G. d'Annunzio” University of Chieti-Pescara, Chieti, Italy; ^4^The College of Healthcare Sciences, James Cook University, Townsville, QLD, Australia; ^5^Faculty of Sports of the University of Porto, Porto, Portugal; ^6^Interdisciplinary Center of Human Performance Studies (CIPER), Faculty of Human Kinetic, University of Lisbon, Lisbon, Portugal; ^7^Department of Health and Kinesiology, Georgia Southern University, Statesboro, GA, United States

**Keywords:** maturation, canoe sprint, young kayakers, 1000 m, time-trial, V*O*_2max_, canoeing and kayaking, ventilatory threshhold

## Abstract

This study aimed to develop a predictive explanatory model for the 1,000-m time-trial (TT) performance in young national-level kayakers, from biomechanical and physiological parameters assessed in a maximal graded exercise test (GXT). Twelve young male flat-water kayakers (age 16.1 ± 1.1 years) participated in the study. The design consisted of 2 exercise protocols, separated by 48 h, on a kayak ergometer. The first protocol consisted of a GXT starting at 8 km.h^−1^ with increments in speed of 1 km.h^−1^ at each 2-min interval until exhaustion. The second protocol comprised the 1,000-m TT.

**Results:** In the GXT, they reached an absolute $\overset{∙}{V}$*O*_2*max*_ of 3.5 ± 0.7 (L.min^−1^), a maximum aerobic power (MAP) of 138.5 ± 24.5 watts (W) and a maximum aerobic speed (MAS) of 12.8 ± 0.5 km/h. The TT had a mean duration of 292.3 ± 15 s, a power output of 132.6 ± 22.0 W and a $\overset{∙}{V}$*O*_2*max*_ of 3.5 ± 0.6 (L.min^−1^). The regression model [TT (s) = 413.378–0.433 × (MAP)−0.554 × (stroke rate at MAP)] presented an *R*^2^ = 84.5%.

**Conclusion:** It was found that $\overset{∙}{V}$*O*_2*max*_, stroke distance and stroke rate during the GXT were not different from the corresponding variables ($\overset{∙}{V}$*O*_2*peak*_, stroke distance and stroke rate) observed during the TT. The MAP and the corresponding stroke rate were strong predicting factors of 1,000 m TT performance. In conclusion, the TT can be useful for quantifying biomechanical parameters (stroke distance and stroke rate) and to monitor training induced changes in the cardiorespiratory fitness ($\overset{∙}{V}$*O*_2*max*_).

## Introduction

Olympic male flat-water kayak competitions comprise 200 and 1,000 m distances. At Rio 2016, medals won within 35.195 to 35.662 s and 211.447 to 213.363 s, respectively, for 200 and 1,000 m. Performance in different competitive distances requires a distinct combination of physiological and anthropometric factors (1). At the adult national and international level, 200 m race time recorded over a flat-water course was significantly correlated to maximal accumulated oxygen deficit and performance during a kayak ergometer adapted Wingate test (1). Besides, upper body strength and some selected anthropometric characteristics (e.g., humerus breadth) significantly correlated with 200 m race performances (1). In young kayakers, on-water performances in both 200 and 1,000 m distances could be predicted (88 and 85%) from a kayak ergometer protocol, using multiple regression, including the maximal oxygen uptake, the maximal aerobic power, and near-infrared spectroscopy derived deoxyhaemoglobin (2). Despite the high explanatory power of the “aerobic-derived” variables included in the statistical model, the referred authors did not explore the relationship between morphological and biomechanical variables and on-water kayak performance, which can also be considered determinants of competitive success (2).

Among the main variables considered in the biomechanical evaluation using kinematic parameters of kayaking, the stroke rate and the stroke distance can be highlighted, with stroke rate being the most common monitored variable by coaches during the competition (3–5).

Special care is needed while working with young athletes concerning maturation since performance can be modulated by morphological and maturation status (6). Studies have shown that kayakers aged between 13 and 16 years are usually early maturers, possessing longer limbs, greater lean body mass and reduced subcutaneous fat, associated with higher stature and weight than non-athlete counterparts (7). Hence, young kayakers are predominantly ecto-mesomorphic (8). The influence of these maturational variables on specific performance still needs to be addressed in conjunction with physiological traits in young kayakers.

Despite the high ecological validity of on-water performance, there are difficulties in monitoring elite athletes in their competitive distance under real conditions. In race situation, the environmental changing conditions could affect performances and their interpretation. Coaches and sport scientists must use valid and accurate instruments with equally valid and reliable protocols to detect training adaptations that could influence performance (9). Ergometers simulate the movement patterns of a sport, allowing access to the physiological responses in a controlled laboratory environment (10). The comparison of ergometer with on-water kayaking performance has concluded that kayak ergometers accurately simulate the physiological demands of short-term high-intensity kayaking (10, 11). The use of ergometers offers advantages in the evaluation of athletes performance in a laboratory context (3–5, 9), bringing advantages of methodological rigor in the control of athletes in the laboratory context.

The study aims first to verify the predictive power of biomechanical, physiological, maturational and the morphological characteristics on a 1,000 m time-trial performance (TT), assessed by a maximal graded exercise test (GXT) in young kayakers. Second, compare biomechanical and physiological parameters between both tests (TT and GXT–$\overset{∙}{V}$O_2max_). The knowledge of the most influential variables affecting the 1,000 m TT in kayak ergometer in young kayakers, could help coaches to interpret relevant physiological and biomechanical variables, assisting in the selection for competitive proposes.

## Methods

### Participants

Twelve (*n* = 12) Portuguese national-level male young sprint kayakers volunteered to take part in this study [mean ± SD; age 16.1 ± 1.1 years (yr), stature 175.0 ± 7.0 cm, siting height 92.5 ± 3.8 cm, weight 63.7 ± 7.1 kg, body fat 6.8 ± 2.1 kg, % body fat 10.7 ± 2.9 %, training experience 2.7 ± 0.7 yr, mean weekly training volume 10.6 ± 2.4 h]. The same experienced researcher performed the anthropometric measurements. The athletes visited the laboratory on two occasions during which they accomplished two experimental protocols (GXT and TT). The athletes were familiar with the kayak ergometer and testing procedures applied. On the first visit, upon arrival at the laboratory, the athletes completed a pre-test questionnaire to characterize the training and food intake in the last 24 h. The questionnaire was adapted from Tanner and Gore (12) and was used to verify if the athletes maintained the same pattern of activity and nutrition before the protocols. The data were analyzed and returned to the athletes, indicating to keep the same nutrition and hydration schedule for the second visit. Briefly the macronutrients distribution was as follows: protein 21.9 ± 6.3%; fat 30.0 ± 8.0%, and CHO 48.1 ± 11.3%. They also completed a symptoms questionnaire to evaluate the health status and perception of the athlete's recovery level. The distance from seat to foot-bar and hand position on the carbon shaft were adjusted individually and maintained constant across the tests. The maturational status was estimated through the maturity offset that predicts years from peak height velocity (PHV), based on anthropometric variables (height, sitting height, weight, leg length) (13). The athletes presented 2.20 ± 1.03 years post-PHV. The legal guardians and the young athletes gave their written consent after receiving a detailed explanation of the purposes and procedures of the study. The local Ethics Committee of the Faculty of Sports Science and Physical Education, University of Coimbra (CE/FCDEF-UC/00292019) approved the procedures.

### Design

The athletes were familiar with the kayak ergometer and testing procedures applied. The testing situations were conducted at least 24 h apart and completed within 10 days. All testing sessions were held in the same period of the day (17:00–19:00 h). The day before testing, athletes were required to avoid fatigue accumulation; all participants were asked to abstain from intense physical exertion and only a light intensity workout 24 h prior to each testing session was allowed.

The anthropometric and body composition assessments were done during the first visit to the laboratory. Body mass was measured using a calibrated scale (Seca 770, Hamburgo, Germany) and stature and sitting height were measured using a standard stadiometer (Harpenden 98.607, Holtain, UK). Body composition was assessed through plethysmography (Bodpod 2006, Concord, California). Both protocols were performed in an air-braked, drag adjustable ergometer designed to assess flat-water kayakers (Kayak-Ergometer Dansprint, Hvidovre, Denmark).

Ergometer drag was adjusted for body mass of each athlete according to manufacturer's instructions to reproduce on-water surface when on the kayak (www.dansprint.com). The same warm-up procedures were implemented before both tests. Briefly, after 10′ of freely chosen light stretching exercises, they paddled for 15′ at an intensity lower than 70% of their estimated HR_max_, during which athletes were asked to perform 5 short sprints of 3″ (4), followed by a passive rest period of 10′. An active recovery on cycle ergometer lasting 15 min with reduced power output [35 watts (W)] at a pace of 60 rpm was performed after each kayak-specific testing protocol. During the graded maximal test and time-trial, speed, stroke rate, distance per stroke, and mechanical power output were continuously measured through the Dansprint software.

The values of temperature (24.9 ± 0.8°C), ambient humidity (44.3 ± 2.1%) and pressure (763.7 ± 1.0 mmHg) were maintained constant during the tests (Oregon Instruments USA). After both tests, blood lactate (La) was determined at 1, 3, 5, and 7 min of recovery from capillary blood samples collected using heparinized ear-lobe tubes for determination of peak lactate value (Lactate Pro®). Ventilatory parameters were monitored breath by breath with a Quark CPET COSMED® gas analyser (Quark-CPET COSMED, Roma, Italy) calibrated before each testing session according to manufacturer's instructions.

### Test Protocols

*Maximal graded test:* Speed started with paddling at 8 km.h^−1^, with increments of 1 km.h^−1^ each 2 min, until exhaustion. The data was averaged in 30 s segments. Additionally, maximum aerobic power (MAP), maximum aerobic speed (MAS), power output, stroke rate and distance per stroke at each intensity were calculated. Before testing, the gas analyser system (Quark CPET, Cosmed, Rome, Italy) was calibrated for room and gas with known O_2_ and CO_2_ concentrations following the manufacturer's recommendations. The determination of ventilatory thresholds (VT_1_ and VT_2_) and $\overset{∙}{V}$O_2max_ followed the criteria proposed by Howley et al. (14). Briefly, VT_1_ corresponds to the last point before the first non-linear increase in both VE and VE/VO_2_. VT_2_ corresponds to the point before the second non-linear increase in both VE and VE/VO_2_, accompanied by a non-linear increase in VE/VCO_2_. Three experienced researchers identified the ventilatory parameters and a fair agreement was obtained. Coefficients of variation (CV) between researchers ranged from 0.3 to 2.7%. The determined intraclass-correlations (ICC) were 0.93, 0.90 and 1 for VT_1_, VT_2_, and $\overset{∙}{V}$O_2max_, respectively, for the values reported by the three researchers. Accordingly, the mean of each of the value were used in the ventilatory data analysis.

*1,000 m time-trial:* The athletes were required to perform the 1,000 m time-trial (TT) in the shortest time, while receiving verbal encouragement to maintain speed as high as possible. The athletes had full access to a visual display of TT variables, such as distance traveled, time, and stroke rate. During the 1,000 m time-trial the same gas analyser system (Quark CPET, Cosmed, Rome, Italy) was used and the peak oxygen uptake ($\overset{∙}{V}$O_2peak_) was determined. The $\overset{∙}{V}$O_2peak_ was defined as the highest continuous 30 s during the exercise bout.

### Statistical analysis

All data are presented as mean and standard deviation values. The normality and the homogeneity of the data were checked using the Shapiro–Wilke and Levene tests, respectively. A priori sample size estimation was computed. For a sample size of 12 subjects a statistical power of β = 0.9 was found in the linear regression approach of 2 predictors, with an ES = 0.7 and α = 0.05. The student's *t*-test for pairwise comparison was used between the variables obtained in the GXT and TT. Effect sized was computed to ascertain magnitude of the difference of the mean trivial (0–0.19), small (0.20–0.49), medium (0.50–0.79), and large (0.80 and greater) (15). If normal distribution was not verified, the non-parametric Wilcoxon test was used. Correlation analysis was conducted with Pearson (or Spearman–rho correlation coefficient, when normal distribution was not observed) while analyzing the data derived from the GXT and performance during the TT. A multiple regression analysis was used to predict TT performance selecting the GXT's variables that were significantly correlated with performance, being entered into backward multiple regression analysis to predict performance with the lowest standard error of the estimate. The statistical analyses were performed using SPSS (version 24.0, SPSS, Chicago, IL, USA) program and *p* ≤ 0.05 determined the statistical significance.

## Results

### Maximal Graded Exercise Test and 1,000 m Time-Trial

The results of the GXT and the physiological, metabolic and biomechanical variables observed during the 1,000 m time-trial are presented in Table 1. The 1,000 m time-trial lasted 292.2 ± 15.0 s. Although the mean values of the same studied variables were not significant (*p* > 0.05), correlations between VO_2peak_, stroke rate and stroke distance obtained during the TT and the corresponding in the GXT were found. Between protocols (GXT and TT) correlation of the mean speed and mean power output also founded (see Table 2). Additionally, we observed significant lower values during TT when compared to those achieved during the GXT, namely MAS (*t* = −3.888; *p* = 0.003) and a medium mean difference (ES = 0.79; CI = −0.385, 1.965) and MAP (*t* = −2.472; *p* = 0.031) with a small mean difference (ES = 0.25; CI = −0.885, 1.387). Higher blood lactate values were observed at the end of the TT when compared to that recorded after finishing the GXT (*t* = −3.793; *p* = 0.003) and a high mean difference (ES = −0.976; CI = −2.173, 0.221).

**Table 1:** Descriptive data, of maximal graded exercise test and 1,000 m time trial (mean ± SD).

**Variable**	**Unit**	**GTX**	**TT**
**Ventilatory threshold 1 (VT**_**1**_**)**
$\overset{∙}{\text{V}}$O_2_	L.min^−1^	1.96 ± 0.37	
$\overset{∙}{\text{V}}$O_2_	mL.kg^−1^.min^−1^	30.91 ±5.34	
% $\overset{∙}{\text{V}}$O_2max_	%	56.56 ± 8.21	
Heart rate	bpm	157 ± 14	
Speed	km/h	9.92 ± 0.79	
% MAS	%	77.86 ± 5.52	
**Ventilatory threshold 2 (VT**_**2**_**)**
$\overset{∙}{\text{V}}$O_2_	L.min^−1^	2.72 ± 0.51	
$\overset{∙}{\text{V}}$O_2_	mL.kg^−1^.min^−1^	42.89 ± 7.36	
% $\overset{∙}{\text{V}}$O_2max_	%	78.00 ± 5.64	
Heart rate	bpm	177 ± 9	
Speed	km/h	11.50 ± 0.67	
% MAS	%	89.98 ± 3.63	
**$\overset{∙}{\text{V}}$O**_**2max**_
$\overset{∙}{\text{V}}$O_2_	L. min^−1^	3.50 ± 0.65	3.02 ± 0.37
$\overset{∙}{\text{V}}$O_2_	mL.kg^−1^.min^−1^	55.00 ± 8.80	47.56 ± 4.09
% $\overset{∙}{\text{V}}$O_2 max_	%		87.23 ± 7.42
$\overset{∙}{\text{V}}$O_2 peak_	L.min^−1^		3.50 ± 0.57
$\overset{∙}{\text{V}}$O_2 peak_	mL.kg^−1^.min^−1^		55.09 ± 7.25
Heart rate	bpm	195 ± 8	182 ± 10
MAS/TT speed	km/h	12.75 ± 0.45	12.34 ± 0.58^*^
MAP/TT power output	Watts	138.47 ± 24.50	132.63 ± 21.98^*^
% MAS	%		96.70 ± 2.84
Stroke rate	str.min^−1^	110.28 ± 9.17	106.60 ± 6.91
Stroke distance	m	1.91 ± 0.12	1.94 ± 0.09
Time	s		292.30 ± 15.00
Lactate	mmol.L^−1^	10.04 ± 2.12	12.43 ± 2.35^*^

*$\overset{∙}{V}$O_2_, oxygen consumption; bpm, beats per minute; MAS, maximum aerobic speed; MAP, maximum aerobic power*.

* *p <0.05*.

**Table 2:** Bivariate correlation between variables of performance and morphological, biomechanical, and physiological parameters.

**Xi (size descriptors)**	**Yi (Performance)**	**IC (95%)**
	***r***	
Age	−0.698^*^	−0.919; −0.322
VO*_2*max*_*	−0.605^*^	−0.848; −0.245
MAS	−0.754^*^	−0.952; −0.484
VT_1_ speed	−0.709^*^	−0.929; −0.280
VT_2_ speed	−0.652^*^	−0.901; −0.193
Stroke rate	−0.719^*^	−0.932; −0.326
MAP	−0.890^*^	−0.965; −0.722

*r, correlation coefficient; VT, Ventilatory threshold; MAS, Maxime aerobic speed; MAP, Maxime aerobic power*.

* *p < 0.05*.

Table 2 displays the significant correlations obtained between athletes' characteristics and physiological/biomechanical variables obtained in the GXT and performance during the TT. Age, $\overset{∙}{V}$O_2max_, MAS, VT_1_, and VT_2_ velocities, stroke rate and MAP displayed significant and negative correlations with time during TT. No morphological and maturational variables were significantly correlated with TT performance.

### Explanatory Regression Model

The multiple regressions enter method analysis revealed that MAP (β = −0.43, *p* = 0.001) and the corresponding stroke rate (β = −0.55, *p* = 0.04) were strong predictors of TT performance. The regression model explained (adjusted *R*^2^) 84.5% of the 1,000 m TT performance [*F*_(2, 12)_ = 30.985 *p* < 0.000].

The resulting predictive equation is as follows:

(1) $$\begin{array}{l} TT\left(s\right)=\;413.378-0.433\times \left(MAP\right)\\ \;-0.554\times (stroke\;rate\;at\;MAP) \end{array}$$

## Discussion and Conclusions

This study aimed first to analyse the predictive value of biomechanical, maturational, morphological characteristics, and physiological parameters determined through a GXT on the 1,000 m TT performance in kayak-ergometer in young national-level kayakers. Second, compare biomechanical and physiological parameters between both tests (TT and GXT–$\overset{∙}{V}$O_2max_). It was found that $\overset{∙}{V}$O_2peak_, the stroke distance and stroke rate during the GXT were not different from the corresponding variables observed during the TT. Besides, the MAP and the corresponding stroke rate were strong predictive factors of 1,000 m TT performance.

Our results showed that $\overset{∙}{V}$O_2peak_ measured during the 1,000 m TT was not different and highly correlated with $\overset{∙}{V}$O_2max_ determined during the GXT. Hence, this study demonstrated that in well-trained young kayakers, cardiorespiratory fitness could be assessed during a time-trial test that mimics the performance attained in flat water. This result is of practical interest since athletes prefer to complete a TT instead of progressive tests. Performance (i.e., time to complete the task) can also be monitored using the former. The agreement between the GXT and TT founded in our study agrees with that obtained in $\overset{∙}{V}$O_2peak_ reached during the incremental test and time-trial in cycling athletes (16). The mean relative $\overset{∙}{V}$O_2max_ value found in our study is very similar to that measured in junior kayakers reported before (2). Besides, the average stroke rate and stroke distance recorded during the TT were also identical to those obtained during the GXT. Therefore, the 1,000 m TT can offer several parameters that are useful in assessing athletes while being easier to implement than GXT. Moreover, TT may be performed more often as a method to control performance without the influence of environmental factors (e.g., wind) (10).

Is well-accepted that in young athletes, the older athletes trend performed better, which could be explained by maturation relative factors. Athletes maturational advanced tend to show better performances as observed in Spanish young canoe sprint athletes which reinforce the need to evaluate age and maturity to interpret the performance in young canoe sprint athletes (8, 17). In our study, we did not observe the association of maturity on the performance due to the homogeneity of the sample that clearly surpass the PHV.

Comparing the results of the GXT and TT, we found differences on the percentage of MAP (4.2%), MAV (3.2%), absolute $\overset{∙}{V}$O_2_ (13.7%), and HR (6.7%). In another study, the model regression analysis showed that the $\overset{∙}{V}$O_2max_ was the best predictor of performance in 1,000 m TT, which is in line with our results (2). A study involving Australian canoe sprint elite athletes showed that the smallest change of 0.3% in 200 m race time could be achieved with a 0.9% change in $\overset{∙}{V}$O_2max_ (18). In longer distance events with a higher aerobic contribution (e.g., 2,000 m), it can be expected that a similar increase will further improve 1,000 m performance in $\overset{∙}{V}$O_2max_. The relative intensity of VT_1_ and VT_2_ found in our study are also in agreement with others (19). Previous research did not report the verified correlation of the velocity at VT_1_ and VT_2_ intensities with that of 1,000 m. These results support the importance of the aerobic-fitness variables as a valuable and useful marker for monitoring longitudinal athlete development and. They could also reinforce that better submaximal (velocity) performance influences the all-out time-trial performance.

The biomechanical variables showed that the athletes with the highest stroke rate and highest mechanical power output performed better in the TT, being able to apply and maintain a power value close to the MAP (17). However, in the study mentioned above conducted on water, the stroke distance and the stroke rate were reported as critical factors for mechanical efficiency and performance in young kayakers (20), whereas stroke distance was not significantly correlated with performance in the current study.

The lactate concentration values found in this study highlights the contribution of the anaerobic energy pathway to kayak TT performance and reinforces the role of the significant contribution of anaerobic energy sources in sprint kayak performance (5). The lactate value at the end of TT is in line with that reported in another study (3). The higher value found at the end of TT could be explained by greater exercising time at high intensity, in the TT, than GXT protocol where the sample remained up to 120 s at similar intensity.

The equation developed in our study showed higher explanatory power than that of Van Someren et al. (21) probably because we added the stroke rate at $\overset{∙}{V}$O_2max_ which is a critical kinematic variable to performance, in conjunction with the maximal aerobic power.

## Conclusion

In conclusion, we found that $\overset{∙}{V}$O_2max_, stroke distance and stroke rate during the GXT were not different from the corresponding variables ($\overset{∙}{V}$O_2peak_, stroke distance and stroke rate) observed during the TT, and that MAP and the corresponding stroke rate were strong predictors of 1,000 m TT performance. Our results confirmed that $\overset{∙}{V}$O_2max_, chronological age, the speed at VT_1_ and VT_2_, stroke rate and power output are significantly correlated with the performance in a 1,000 m time-trial. This study demonstrated that in well-trained young kayakers, cardiorespiratory fitness could be assessed during a time-trial test that mimics the actual performance attained in flat water. The final lactate confirmed the high contribution of the lactic anaerobic pathway (5). Therefore, coaches can use these variables ($\overset{∙}{V}$O_2max_, chronological age, the speed at VT_1_ and VT_2_, stroke rate and power output) as performance markers when monitoring their athletes with regards to predictive factors of sport-specific performance. Programming training to improve these variables may therefore also improve 1,000-m kayaking performance.

## Practical Implications

TT testing on a kayak ergometer may be a suitable and preferable alternative to graded exercise testing for determining $\overset{∙}{\text{V}}$O_2max_, the stroke distance and stroke rate among young, national-level kayakers due to its practicality and the ability to mimic actual performance. Findings from the current study suggest that $\overset{∙}{\text{V}}$O_2max_, chronological age, the speed at VT_1_ and VT_2_, stroke rate and power output are significant predictors of 1,000 m kayaking performance. Therefore, coaches can use these variables as performance indicators when monitoring their athletes with regards to predictive factors of sport-specific performance. Programming training to improve these variables may hence also improve 1,000 m kayaking performance.

## Limitations

The main limitation of the study was the small sample size. However, the athletes in the sample, are representative of the Portuguese national age group in flatwater. Unfortunately, we are not possible to research the ecological environment due to the equipment limitations. Nevertheless, the laboratory equivalent conditions in the applied protocols seem to be an appropriate research option with some advantages.

## Data Availability Statement

All datasets generated for this study are included in the article/supplementary material.

## Ethics Statement

The studies involving human participants were reviewed and approved by Local Ethics Committee of the Faculty of Sports Science and Physical Education, University of Coimbra (CE/FCDEF-UC/00292019) approved the procedures. Written informed consent to participate in this study was provided by the participants' legal guardian/next of kin.

## Author Contributions

AC and LR conceived the experiment. AC, MM, FN, and LR designed and conducted the experiment. AC, FN, FA, AF, AD, MM, and LR analyzed the data, performed statistical analysis, and primary responsibility for the final content. All authors have read and approved the manuscript.

## Conflict of Interest

The authors declare that the research was conducted in the absence of any commercial or financial relationships that could be construed as a potential conflict of interest.
